# Oxidative Stress in Obesity

**DOI:** 10.3390/antiox11040639

**Published:** 2022-03-26

**Authors:** Ernesto Martínez-Martínez, Victoria Cachofeiro

**Affiliations:** 1 Departamento de Fisiología, Facultad de Medicina, Universidad Complutense de Madrid–Instituto de Investigación Sanitaria Gregorio Marañón (IiSGM), 28040 Madrid, Spain; 2 Ciber de Enfermedades Cardiovasculares (CIBERCV), Instituto de Salud Carlos III, 28220 Madrid, Spain

Obesity is defined by the World Health Organization (WHO) as abnormal or excessive fat accumulation that presents a health risk. Obesity has reached epidemic proportions, with more than 4 million people dying due to being overweight or obese. Data on the prevalence of overweight and obesity are alarming, showing that around 40% of adults are overweight and more than 13% are obese, and over 39 million children under 5 years old are overweight or obese. Obesity is a major risk factor for different diseases, such as cardiovascular diseases, cancers, neurological alterations, respiratory problems, and musculoskeletal disorders. For all of these reasons, it is necessary to understand the mechanisms involved in obesity under different pathologies and identify new molecular targets and pharmacological approaches to prevent adverse outcomes.

Oxidative stress is a common pathway that links obesity with related complications. Oxidative stress is defined as an imbalance between antioxidants defenses and free radical production. This oxidative environment could be one of the main determinants that trigger other mechanisms involved in tissue damage, such as inflammation, extracellular matrix overproduction, endoplasmic reticulum stress activation, and autophagic flux disruption.

This Special Issue highlights the detrimental effects of oxidative stress in different scenarios in the context of obesity. In this sense, it has been observed that young patients with metabolic syndrome, with the highest levels of oxidative stress, demonstrate a decrease in superoxide dismutase activity, thereby showing the relevance of impaired activity of this antioxidant defense in the participation of the oxidative environment. Moreover, this study shows that obesity and insulin resistance are the two main components of metabolic syndrome associated with oxidative stress, suggesting the close relationship between oxidative stress and excessive fat accumulation [1]. In the same way, another study showed that a reduction in body weight due to a moderate caloric restriction for 8 weeks in obese patients promoted an increase in antioxidant defense and a decrease in oxidative stress markers. These changes were also accompanied by an improvement in glucose tolerance and, therefore, a decrease in insulin resistance [2]. The study performed by Lejawa M. et al. focused on the association between telomere length, oxidative stress, and obesity, showing a reduction in telomere length in young metabolically unhealthy obese patients. The study also shows that total oxidation status, total antioxidant capacity, and telomere length were significantly related in these patients [3]. Losing body weight also showed benefits in cardiovascular diseases such as subclinical atherosclerosis. One year after bariatric surgery, patients showed a decrease in superoxide anion production and increased antioxidant defense in leukocytes, which were associated with improvement of different markers of atherosclerosis and metabolic outcomes [4]. Obesity and oxidative stress link are also related to vasculature abnormalities. The study performed by González-Amor M et al. demonstrated that, in obese patients and an experimental model of obesity, G protein-coupled receptor kinase 2 (GRK2) emerges as a potential therapeutic target in the development of endothelial dysfunction observed in this pathological situation. This suggestion is based on the fact that GRK2 is associated with inflammatory markers in obese patients, and its downregulation from myeloid cells in obese animals ameliorates inflammatory and oxidative stress markers and prevents the impairment of endothelium-dependent vasodilator responses induced by perivascular adipose tissue in obese mice [5].

Oxidative stress is also related to autophagy dysregulation. The concomitant oxidative stress and autophagy suppression generate an obesogenic environment [6]. Biopsies of visceral adipose tissue from obese patients revealed that treatment with metformin in type 2 diabetic patients decreased inflammation and oxidative stress markers, facts that were accompanied by an improvement in autophagy flux [7]. At the hepatic level, obesity promotes lipid peroxidation, oxidative stress, and autophagy flux impairment in mice fed a high-fat diet combined (or not) with a high-sucrose diet. These alterations in the autophagy flux could be relevant in liver diseases such as nonalcoholic fatty liver disease [8]. Authors in the study observed that, in this scenario, the alterations mentioned were in the absence of mitochondrial alterations, the main source of free radicals [8]. However, mitochondrial oxidative stress can play a relevant role in the cardiac damage associated with obesity since treatment with a specific mitochondrial antioxidant (MitoQ) in obese rats was able to prevent cardiac alterations characterized by cardiac fibrosis. In addition, the treated obese rats did not develop endoplasmic reticulum stress compared with untreated obese animals, showing an interaction between mitochondrial oxidative stress and endoplasmic reticulum stress in the production of extracellular matrix proteins [9]. This mitochondrial oxidative stress participation was confirmed in another study in which treatment with another mitochondrial antioxidant (MitoTempo) was able to reverse dysbiosis observed in obese animals. This effect of the mitochondrial antioxidant in the gut microbiota was accompanied by an improvement in the cardiac fibrosis and insulin resistance observed in the obese animals, illustrating the relevance of mitochondria as one of the main sources of free radicals and a potential therapeutic target for treating the complications of obesity [10].

This Special Issue illustrates the role of oxidative stress in different alterations and changes associated with obesity and shows the potential beneficial effects of antioxidant treatment for obesity complications. In the same way, another experimental study reported the favorable effects of dietary antioxidant compounds on body weight, mitochondrial alterations, and adipose tissue remodeling in obese mice [11]. However, although numerous data support the beneficial effects of antioxidants, this Special Issue includes a study that showed that the administration of pure polyphenols as a food supplement has detrimental consequences on insulin resistance and kidney and liver fibrosis [12]. These conflict data support the need for more clinical and experimental studies to improve the understanding of the role of oxidative stress on obesity and develop new therapeutic strategies against this complicated pathological scenario.
